# Mechanical properties and acoustic emission characteristics of deep hard coal after segmented high-temperature treatment

**DOI:** 10.1038/s41598-022-26403-8

**Published:** 2023-01-20

**Authors:** Chun Wang, Xin-ru Li, Lu-ping Cheng, Dong-ping Jiang, Zu-qiang Xiong, Bin-bin Lei, Pan-long Zhang, Shuai-fei Zhan

**Affiliations:** 1 Sinosteel MaAnShan General Institute of Mining Research Co., LTD, Maanshan, 243000 Anhui China; 2 School of Energy Science and Engineering, Henan Polytechnic University, Jiaozuo, Henan 454003 China; 3 Collaborative Innovation Center of Coal Work Safety and Clean High Efficiency Utilization, Jiaozuo, Henan 454003 China

**Keywords:** Energy harvesting, Fossil fuels

## Abstract

Based on engineering background that local heating of coal seam is uneven due to underground coal gasification, coal-bed gas exploitation via heat injection, spontaneous combustion of coal seam, etc., segmented heating coal sample was used to simulate coal seam under uneven heating condition, and experimental study on mechanical behaviors of coal sample after segmented heat treatment at high temperatures was conducted. Test results show that temperature at 100 °C ~ 400 °C did not reach ignition temperature of deep hard coal for the experiment and was not enough to change main ingredients of coal sample, which less affected compression strength, elastic modulus, acoustic emission behavior of coal sample. Although compaction stage-elastic stage-plastic stage-broken stage appeared in compression stress–strain curve of coal sample, height increase led to decrease of compression strength, elastic modulus of coal sample, cumulative amplitude and ringing count for acoustic emission in the form of power function. Meanwhile, it is found that final failure modes of coal sample after segmented heat were mainly shear failure and separation failure and friction mixed failure was secondary. In addition, influence of heating temperature at 100 °C ~ 400 °C on failure modes of coal sample was small. However, height increase in the heating section of coal sample made shear failure surface gradually move to the heating section and separation failure surface moved with the change of contact surface position between heating section and non-heating section. Furthermore, the integral failure degree of coal sample was more serious. Finally, based on variation behaviors of acoustic emission parameter for coal sample after segmented heating, inversion formula on acoustic emission parameter for strength of coal sample was discussed and verified via experimental result of coal sample with different segmented heat height after heating treatment at 200 °C.

## Introduction

Coal is an essential basic energy source in China. With the consumption of coal resources, its mining depth and difficulty are increasing, accompanied by higher ground temperature. At the same time, new technologies are becoming increasingly mature, such as underground coal gasification and coalbed methane heat injection exploitation proposed for mining the residual coal, reducing transportation costs and efficiently recovering energy^1,2^. In the mining process of coal resources under a different high-temperature environment, the main difficulty was the uneven heating of coal seam, which will lead to the difficulty in predicting the germination, expansion, and penetration of internal cracks, and ultimately result in the difficulty in predicting the subsidence law of overburden rock, the displacement direction of the coal seam, and even the stability of surrounding rock of mining shaft. Therefore, it is urgent to explore the mechanical properties, acoustic emission characteristics, and corresponding failure modes of coal seams at different heating temperatures and locations.

As for the deformation characteristics, compressive strength and elastic modulus of coal samples after high temperature, scholars at home and abroad have conducted a lot of researches, For example analyzing the compressive strength and elastic modulus of anthracite coal after high temperature, it was founded that their values at 500 °C dropped to about 15% of that at room temperature, indicating that the thermal damage produced by high-temperature treatment led to serious deterioration of the overall structure of anthracite coal^3,4^. Some scholars^5–7^ also explored the deformation characteristics of anthracite bodies under thermo-mechanical coupling and found that 400 °C ~ 450 °C was the critical temperature range for the transformation of anthracite bodies from brittle to ductile. By studying the creep characteristics and internal pore evolution laws of gas coal at high temperature, it was found that the gas coal entered the creeping stage in a short time at 400℃, and both the internal pore volume and deformation increased during the pyrolysis reaction of gas coal^8–10^. Yu et al.^11^ and Wu et al.^12^ explored the deformation and structural characteristics of lignite within 400 °C, and concluded that the porosity of lignite increased and then decreased with increasing temperature. Some scholars analyzed the effect of temperature on the mechanical and deformation characteristics of coal containing methane, and found that the higher the temperature, the lower the strength and the larger the deformation modulus of the coal sample during the process of unloading the surrounding pressure. Meanwhile, it was also revealed that the coal body was prone to destabilization failure and gas burst under high-temperature environment^13–15^. By exploring the evolution law of crack structure of coal body under temperature shock and its fracture mechanism of internal microscopic cracks, it can be concluded that temperature shock leads to the expansion and widening of internal cracks of coal body, and the forms of germinating cracks mainly include intergranular fracture, transgranular fracture, and reticular fracture^16–18^. There are also some studies on the acoustic emission characteristics of the coal body during the compression process. Some studies^19–21^ have concluded that the parameters such as the cumulative amplitude of acoustic emission and ringing count of hard coal increased and then decreased with the increase of temperature after high-temperature action. By heating the surface of coal samples, Su et al.^22^ and Jiang et al.^23^ found that the faster the temperature change, the more active the acoustic emission, and the more surface cracks produced. Some scholars studied the acoustic emission evolution characteristics of the protruding coal body in the loading process and found that the acoustic emission intensity of raw coal was much larger than that of briquette, and there was a sudden increase in acoustic emission activity of raw coal, while briquette was relatively stable^24–27^. Other scholars^28–30^ explored the acoustic emission characteristics of coal bodies under different water content conditions and found that the acoustic emission event count rate and cumulative counts of coal bodies decreased with the increasing water content. Given the failure mode of coal and rock mass under high temperature, scholars^31–33^ have tried to use a variety of methods to study. By studying the failure pattern of rocks under different high temperatures it can be found that the failure was transformed from brittleness to ductile brittleness, and the failure degree intensified with the increase of temperature. Li et al.^34–36^ explored the fragmentation characteristics of the coal–rock-like combined body under impact load and concluded that the combinations mostly showed splitting failure, and the failure surface was relatively flat. Some studies^37–40^ have found that the coal body was damaged first when the coal–rock combined body was destroyed under compressive load, and the expansion of cracks might lead to the failure of the rock body.

Based on the above studies, the deformation, strength, acoustic emission, and other characteristics of coal bodies under high temperatures have attracted the attention of scholars. Various methods have been used to carry out research, and considerable results have been obtained. As for the failure mode of coal rock at high temperature, the current research still stays in hard rocks, and the research on the failure mode of soft rocks, such as coal, is inadequate. However, the study also involved the failure mode of the coal–rock combined body and concluded that the coal body was destroyed first during the loading process, which may lead to the failure of the rock body. It provided a theoretical reference for the treatment of the overburden strata in coal mining. At present, due to the emergence of new technologies such as underground coal gasification and coalbed methane mining through injecting thermal, coal seams need to be heated, which led to the phenomenon of uneven heating of coal seams. Therefore, to make up for the lack of theoretical basis of the surrounding rocks control during the mining of unevenly heated coal seams, it was necessary to carry out the study of mechanical properties and acoustic emission characteristics of coal stratified heating.

## Coal sample preparation and test scheme

### Coal sample preparation

The coal samples for the test were taken from a coal mine in Xinjiang, and the coal seam was buried at a depth of approximately 400 m. To ensure the physical and mechanical properties of the prepared coal samples were more relatively consistent, coal blocks with a larger size, better homogeneity, and fewer cracks were selected from the same site of the working face, and then wrapped with plastic film and transported to the rock sample processing laboratory. According to the processing standard of the coal sample for testing, the coal block was processed into a standard cylindrical coal sample with a diameter of 50 mm, a height of 100 mm, and the non-parallelism and non-perpendicularity of the two end faces were less than 0.02 mm by three processes of core drilling, cutting and grinding. Finally, the density of coal samples was determined by the volume product method, and coal samples with similar density, namely about 1.35 kg/cm^3^, were selected to carry out the loading mechanical tests after segmented high-temperature treatment.

### Segmented high-temperature treatment

**Figure 1:**
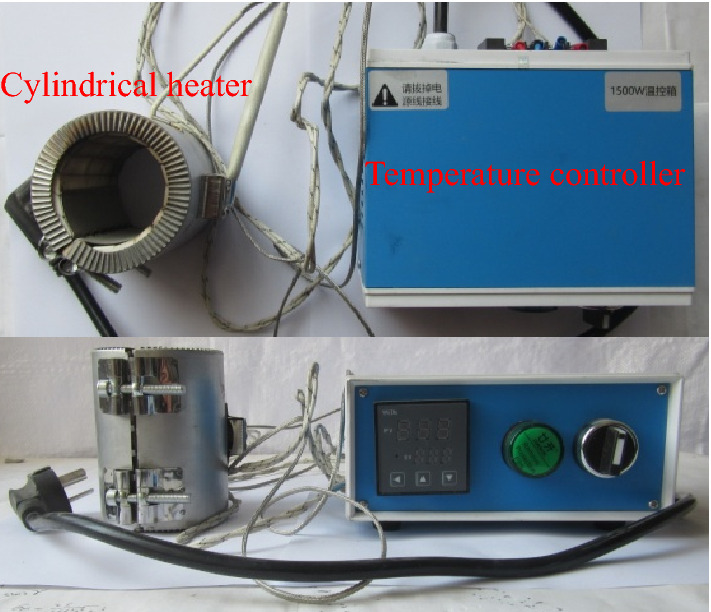
Segmented heating device for coal sample.

Standard coal sample was segmented to be heated by a self-prepared segmented coal sample heating device, mainly composed of a temperature controller and a cylindrical heater, as shown in Fig. 1. The temperature controller was used to control the segmented heating rate and the final temperature. And the function of the cylindrical heater was to realize the control of segmented heating of the cylinder coal sample.

**Figure 2:**
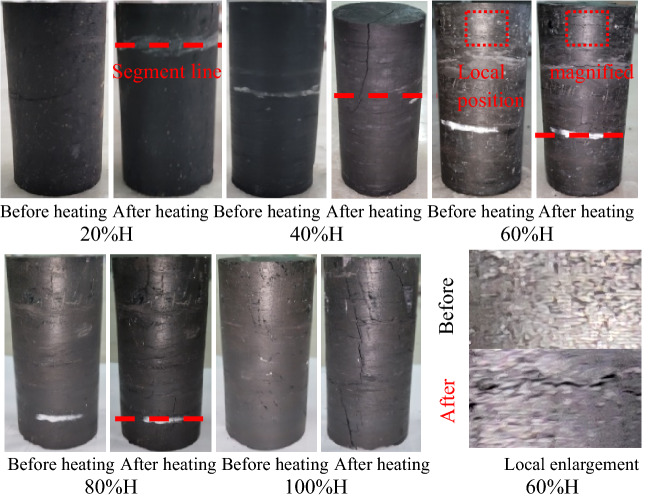
Comparison diagram of partially treated coal samples (Heating temperature is 300 °C) (Note: Before in the local enlargement means before heating; After means after heating).

Before segmented heating of coal samples, sectional lines were marked according to 20%, 40%, 60%, and 80% of the height of coal samples. Then coal sample section to be heated was fixed inside the circular heater and buried in fine quartz sand to prevent spontaneous combustion of the coal sample by contacting with air. Quartz stone is treated with high temperature heating, the purpose of the process is to remove the moisture inside quartz stone. After that, the temperature of the thermostat was set and the heating was powered on. The heating rate was calculated to be 20 °C/min. When the temperature of the thermostat reached the design value and was maintained for 0.5 h to ensure that the coal sample in the heating section was evenly heated, the heater was powered off to lower the temperature, and the coal sample was removed when it cooled down to room temperature. According to the test needs, the segmented heating treatment was carried out in five gradients of 20%, 40%, 60%, 80% and 100% of the coal samples height, and the temperature of the heating treatment was set at four levels of 100 °C, 200 °C, 300 °C, and 400 °C. The segmented heating temperature was determined by combining the following two points. One was that the ignition point of the coal used in the test was 600 °C. Another point was the gradient that the temperature decreased with increasing distance from the source of heat. The comparative graphs of the partially treated coal samples are shown in Fig. 2.

### Test scheme

Underground coal gasification, coalbed methane mining through injecting thermal, and spontaneous combustion of coal seam can cause local uneven heating of coal seam, which changes their physical and mechanical properties and acoustic emission characteristics. Based on the engineering situation of coal seam heating and considering both the temperature and the heated location, coal samples were heated at different temperatures to simulate the high-temperature environment of the coal seam, and the high-temperature effect on the local position of the coal seam was simulated by the partially heating segmented coal samples. On this basis, mechanical loading tests were carried out after segmented high-temperature treatment. The test system mainly consisted of RMT-150B electro-hydraulic servo rock mechanics testing machine and DS-5 8-channel acoustic emission monitoring and analysis device with a threshold value of 50 dB. The test was carried out by arranging the acoustic emission sensors at the dividing line between the heated and unheated sections of the coal sample and applying axial load at a loading rate of 0.002 mm/s. The specific test scheme is shown in Table 1.

**Table 1:** Loading mechanical test scheme after segmented high-temperature treatment.

Rock sample number	Height H/mm	Diameter D/mm	Density ρ/(g/cm^3^)	Heating temperature T/℃	Segmented height H_f_/mm
BF1	99.92	49.24	1.35		
BF2	99.84	49.21	1.36		
BF3	99.88	49.27	1.45		
F1-1	99.69	49.21	1.33	100	20%H
F1-2	99.86	49.35	1.38	200	
F1-3	100.10	49.25	1.37	300	
F1-4	99.90	49.30	1.32	400	
F2-1	100.04	49.19	1.38	100	40%H
F2-2	99.69	49.17	1.33	200	
F2-3	99.97	49.23	1.30	300	
F2-4	99.84	49.19	1.33	400	
F3-1	99.85	49.25	1.44	100	60%H
F3-2	100.07	49.29	1.42	200	
F3-3	99.53	49.19	1.33	300	
F3-4	100.10	49.15	1.33	400	
F4-1	100.00	49.25	1.37	100	80%H
F4-2	100.07	49.22	1.31	200	
F4-3	100.09	49.23	1.35	300	
F4-4	99.92	49.23	1.41	400	
F5-1	100.06	49.19	1.35	100	100%H
F5-2	99.91	49.28	1.30	200	
F5-3	99.92	49.22	1.36	300	
F5-4	99.71	49.15	1.41	400	

## Mechanical properties of coal samples after segmented high-temperature treatment

### Deformation characteristics

**Figure 3:**
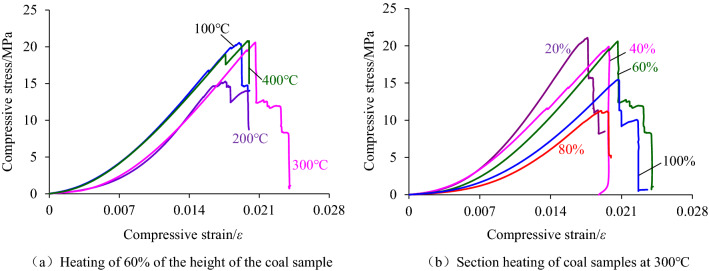
Compression stress–strain curve of coal sample after segmented high-temperature treatment.

The structure and composition of coal samples are prone to deteriorate in a high-temperature environment, especially the stratification phenomenon of coal samples caused by segmented high-temperature treatment. That is, the internal structure and composition of coal samples in high temperature heated section and unheated section are different. To explore the deformation characteristics of coal samples under segmented high-temperature heating, the compressive stress–strain curves of coal samples are listed in Fig. 3. The curves consist of two conditions: the same segmented heating height with different temperatures and the same temperature with different segmented heating heights.

The deformation curves in Fig. 3 reveal that the initial stages all show a concave trend, indicating that the internal cracks of the rock samples were compressed at the initial loading stage. Further comparing the compressive strains corresponding to the end of the concave section under the curve in Fig. 3a and b, it can be found that the latter was slightly larger than the former, This phenomenon indicates that compared with the temperature, the height of the segment was more likely to promote the germination and expansion of microcracks inside the rock sample during heating. The reason was that the delamination occurred at the interface between the heated and unheated segments, which led to the relative increase of strains generated in the coal sample at the early stage of loading. Subsequently, the curve produced a steep drop after a short upward concave section, which can be considered as a small yield deformation of the coal sample that can be produced and eventually produced brittle failure. According to the above analysis, both the height of heating section and heating temperature did not change the nature of coal sample deformation. That is to say, the coal sample deformation still went through four stages (i.e. compaction-elasticity-plasticity-failure), but the segmented heating was more likely to cause stratified cracks inside the coal sample.

### Evolution law of elastic modulus

**Figure 4:**
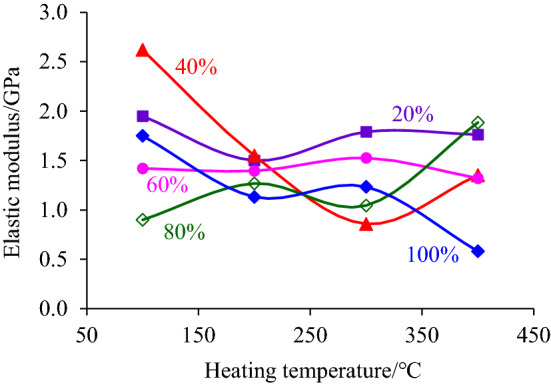
The change rule of the elastic modulus with the heating temperature increasing.

**Figure 5:**
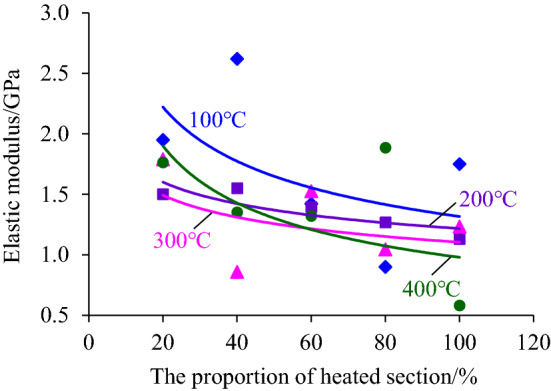
The change rule of the elastic modulus with the increase of the heating segment height.

The slope of the linear segment of the compressive stress–strain curve of the coal sample after intercepting the segmented high-temperature treatment was calculated and the corresponding elastic modulus was obtained. Then the law of its variation with the increase of heating temperature and height of the heating segment was analyzed, as shown in Figs. 4 and 5.

From Fig. 4, it can be found that the elastic modulus of coal samples does not change much at the temperature from 100 to 400 °C, and the overall trend develops nearly horizontal. It can be seen from Fig. 4 that the elastic modulus of coal samples does not change much at the temperature of 100 °C ~ 400 °C, but generally develops in a nearly horizontal trend. In particular, the elastic modulus–heating temperature curve, in which the height of the heating section accounts for 20% and 60% of the height of the coal sample, indicates that the temperature below 400 °C is not enough to change the elastic deformation properties of the coal sample.

The reason is that the temperature of 400 °C did not reach the ignition point of the hard coal used in the test, and the main composition of the coal sample was not changed during heating. However, the elastic modulus decreases or increases with the increase of heating temperature, such as when the height of heating sections accounts for 40% and 80% of the height of the coal sample. The reason for this discrete phenomenon can be attributed to the difference in the original structure inside the coal sample. During heating, water evaporation in the coal sample leads to the enhancement of the elastic deformation property, while the expansion of the original fissure leads to the weakening of the elastic deformation property of the coal sample, which is finally reflected in the local increase or decrease of the elastic modulus.

By fitting the relationship between the elastic modulus and the proportion of the heating segment height, it is found that the elastic modulus decreases with the increase of the proportion of the heating segment to the coal sample height, as shown in Fig. 5. Although the high temperature of 400 °C did not reach the ignition point of the hard coal used in the test and can not change its nature, the heating still promotes the evaporation and dispersion of water and gas escape of the coal samples in the heated section. Also, it caused the separation of the coal samples in the heated section and the coal samples in the unheated section, resulting in local microcracks. The higher the height of the heating segment was, the more moisture and gas were dissipated inside the coal sample, and the local microcracks were relatively increased, which led to the corresponding weakening of the elastic properties of the coal sample, reflected in the elastic modulus of the coal sample decreases with the increase of the height of the heating segment.

### Strength characteristics

**Figure 6:**
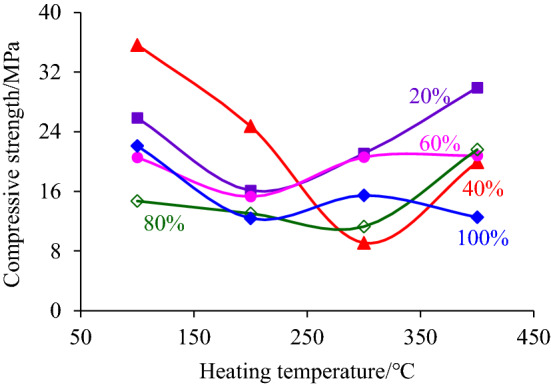
The change rule of compression strength with the heating temperature increasing.

**Figure 7:**
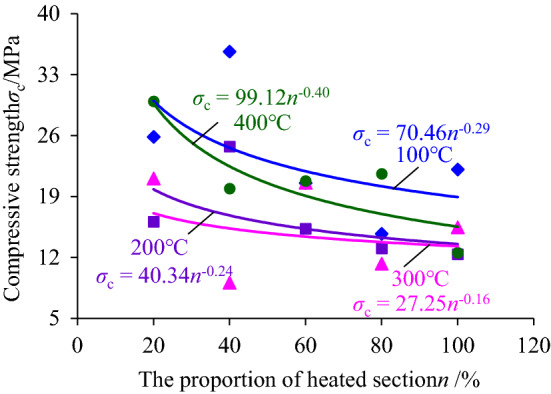
The change rule of compression strength with the increase of the heating segment height.

In order to explore the influence of heating temperature and height of heating segment on the resistance of deep hard coal to external loads, the peak compressive stress of compression stress–strain curve, i.e., the axial compressive strength of coal sample, was extracted and its change rule with the increase of heating temperature and heating segment height was analyzed, as shown in Figs. 6 and 7 respectively.

Figure 6 shows that with the increase of heating temperature, the compressive strength of the coal sample does not change much when the height of heating segment accounts for 60% and 100% of coal sample height, indicating that the temperature between 100 and 400 °C has little influence on the strength of the coal samples. Although the temperature of 100 °C ~ 400 °C will lead to changes in the internal structure of coal samples, such as crack widening, water evaporation, gas escape, etc., the main component of coal samples was not changed. When the axial load was applied, the cracks in the separation layer were gradually compacted to restore the original ability to resist external loading, which was reflected in the horizontal development trend of axial compressive strength with the increase of temperature. In Fig. 6, there is also a phenomenon that the axial compressive strength decreases and then increases with the increase of heating temperatures, such as when the height of the heating segment is 20%, 40%, and 80%. The reason is that the gas inside the coal sample did not escape quickly through the original cracks when heating, and some of the cracks produce axial growth under gas pressure inside the coal sample, leading to the decreasing compressive strength of the coal samples. When the temperature was high, the gas inside the coal sample was more active and could quickly protrude along the original cracks, which has less influence on the original cracks, and the ability of the coal sample to resist external loading, thus giving the illusion of increasing the axial compressive strength. In summary, it can be concluded that the treatment temperature of 100 °C ~ 400 °C has little influence on the axial compressive strength of deep hard coal.

In Fig. 7, by fitting the relationship between the axial compressive strength of coal samples and the proportion of the height of the heating segment, it is found that the compressive strength decreases with the increase of the proportion of the height of the heating segment to the coal sample in a power function trend. When the heating section was heated, it will lead to the generation of delaminated cracks at the contact surface with the unheated segment, and the degree of its deterioration increase with the increase of the heating segment, meanwhile, the higher the height of the heating section was, the larger the proportion of the original cracks inside the coal sample will be disturbed, and although the variation of the main constituents of the coal body and the original cracks was very small, the cumulative amount will increase to a certain extent. The synergistic effect of the above two aspects eventually led to a decrease in the ability of the coal sample to resist the axial compression loading, i.e., the compressive strength showed a power function trend of decreasing with the increase of the proportion of heating section.

## The change rule of the strain corresponding to the compressive strength

**Figure 8:**
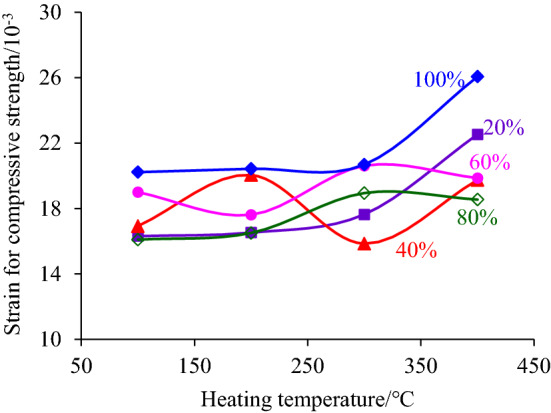
The changing law of the strain corresponding to the compressive strength with the increase of heating temperature.

**Figure 9:**
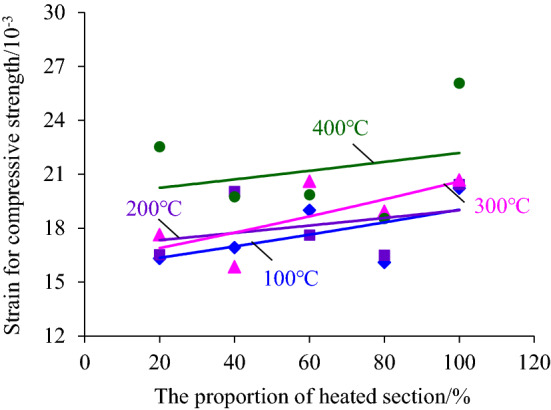
The changing law of the strain corresponding to the compressive strength with the increase of the heating segment height.

In order to study the overall deformation capacity of coal samples before peak stress when coal samples were treated at high temperature in sections and then subjected to axial compression load, the strain corresponding to the compressive strength was selected for analysis. Figures 8 and 9 respectively show the changing law of the strain corresponding to the compressive strength with the increase of heating temperature and height of heating segment.

In Fig. 8, it is shown that the strain corresponding to the compressive strength developed in general with a nearly horizontal trend with the increase of heating temperature, especially when the height of the heating segment was 60% and 80% of the coal sample height, but there was also an abrupt increase of strain, such as the compressive strength corresponding to the strain of the coal sample after high-temperature treatment at 400 °C increased abruptly when the height of the heating segment was 20%, 40% and 80% of the height of the coal sample. The above phenomenon indicated that the temperature between 100 and 400 °C did not play a decisive role in the deformation ability of the coal sample before the peak stress because the ignition point of the hard coal is not reached and thus main composition and structure of the coal sample did not change. The sudden increase in strain corresponding to peak stress after the 400 °C high-temperature treatment was due to the generation of separation cracks at the contact surface between the heated and unheated sections, which led to the increase in strain at the compaction stage of the coal sample during deformation. Therefore, it can be assumed that the high temperature of 400 °C also did not essentially change the deformability of the coal sample.

The higher the height of the heated section was, the more serious the water loss inside the coal sample and the more heat energy was transferred to the unheated section, resulting in a greater chance of expansion of the ordinary microcracks inside the coal sample. Furthermore, the number and width of separation cracks produced by the coal seam increase with the higher height of the heated segment. Therefore, the sample strain corresponding to the compressive strength develops in an increasing trend with the increase of the heating segment height, as shown in Fig. 9. Figure 9 also shows that the increasing trend line is flat and the dispersion of the data is large, which reflects that although the height of the heating segment improves the deformation ability of the coal sample to a certain extent, it does not change the deformation nature of the coal sample, i.e., the coal sample still has a strong brittle nature.

## Acoustic emission characteristics of coal sample after segmented high-temperature treatment

### Acoustic emission characteristics during compression

**Figure 10:**
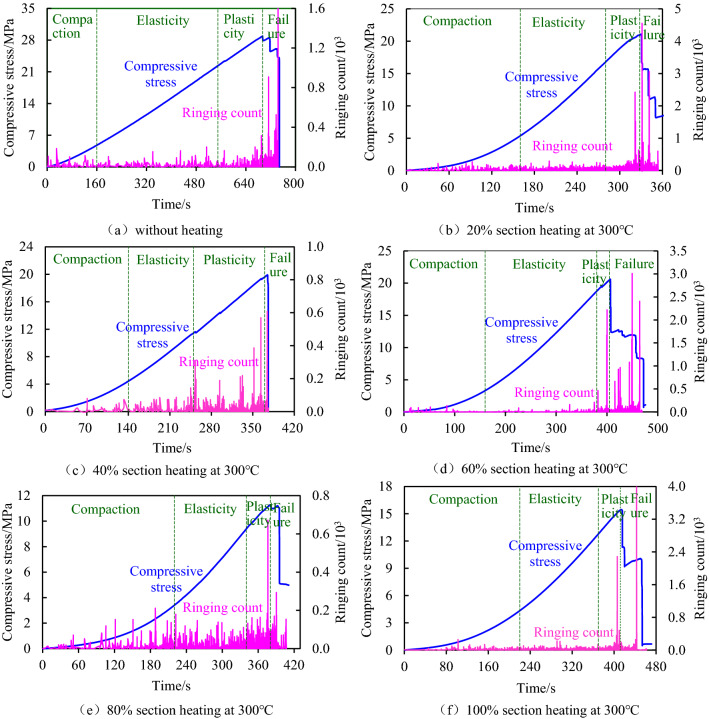
Acoustic emission characteristics of coal samples under the axial load after segmented high-temperature treatment.

Acoustic emission characteristics could reveal the internal fracture development of coal sample subjected to axial load after segmented high-temperature treatment to a certain extent, so as to reflect the degree of damage and failure of coal sample. The evolution law of the strength, elastic modulus and compressive strength corresponding to the strain of loaded coal sample after segmented high-temperature treatment, it could be seen that the temperature of 100 °C ~ 400 °C had no obvious influence on the physical and mechanical properties of coal sample, but the segmented heating height played a major role in weakening the ability of coal sample to resist axial load. Based on this, the variation law of acoustic emission ringing count when the unheated and segmented heating coal samples were subjected to axial compression load was discussed. The damage history of coal samples was revealed, as shown in Fig. 10.

Figure 10 showed that the compression stress and ringing count developed in a segmented manner with the increase of the loading time. By combining the change characteristics of the compression stress–strain curve, the curve could be divided into four stages: compression stage, elastic stage, yield stage, and failure stage. During the process of applying axial load, the coal sample first went through the compaction stage, that was, the micro-cracks inside the coal sample were gradually compacted, and the ringing count corresponding to this stage was small, indicating that the acoustic emission activity was weak. Then the compressive stress-time curve developed approximately in a straight line, namely, the coal sample entered elastic deformation stage, and the ringing count corresponding to this stage increased slightly. The reason was that although the coal sample mainly has elasticity deformation at this stage, there was also the expansion of micro-cracks instability at the local position, which led to the fracture of the local micro coal body and produced a certain amount of acoustic emission, increasing ringing count. Then, the coal sample entered the yield stage, at this stage, the ringing count increased steeply and acoustic emission was active, revealing that the expansion rate of internal micro-cracks increased rapidly during the coal sample yield. Finally, the coal sample entered the failure stage, in which the ringing count was the largest and the acoustic emission activity was the most active. The reason was that macroscopic failure occurred in the coal sample, cracks were rapidly penetrated and coal body fractures were frequent.

Further analyzing the development history of ringing count under the two conditions of non-heating and segmented heating, it could be obtained that when no heating, the compression section was shorter, and the elastic section was longer. Besides, the value of ringing count in the failure stage was the largest, while the increase of ringing count in the yield stage was not obvious. However, it was the opposite for segmented heating. The time corresponding to the compacting stage was relatively long, and the larger the segment height, the longer the corresponding time. The compaction time corresponding to the 80% section heating height and 100% section heating height was the longest. Although the ringing count of the failure stage was also the largest, the ringing count of the yield stage increased more than that of the elastic stage. The reason was that the water and gas in the internal cracks of the heating section escaped from the coal body during the segmented heating, and the separation cracks occurred at the interface of the heating and non-heating sections, which led to the increase of compressible space. As for the yield stage, the cracks generated by heating produced fractures and even proliferated axial stern cracks, leading to a relative increase in the total number of cracks in the coal sample. This mechanism was reflected in the large increase in the corresponding ringing count and relatively active acoustic emission activities in the yield stage.

### Variation characteristics of acoustic emission related parameters

**Figure 11:**
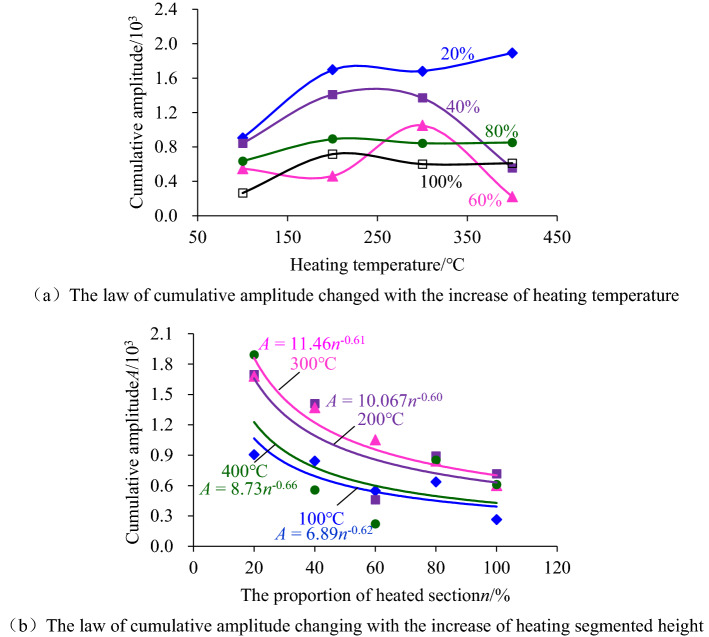
The change rule of accumulated amplitude of acoustic emission.

**Figure 12:**
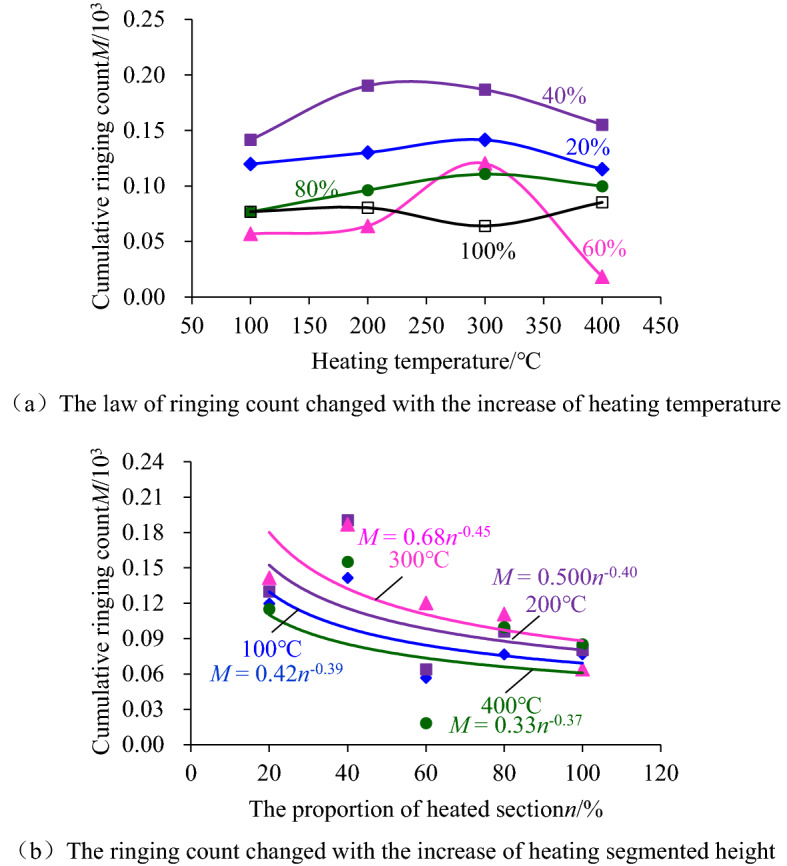
The change rule of cumulative ringing count with the increase of the heating segment height.

The characteristic parameters of acoustic emission could reflect the deformation and failure of coal sample during the loading. Therefore, analyzing the cumulative value of acoustic emission characteristic parameters could indirectly reflect the damage and failure history of the coal sample. In order to explore the characteristics of cumulative values of acoustic emission parameters corresponding to the critical state of coal sample failure, the cumulative acoustic emission amplitude and ringing count corresponding to coal sample failure were selected for analysis. Figures 11 and 12 showed the variation law of cumulative amplitude and cumulative ringing count for different heating temperatures and segmented heating heights, respectively.

As shown in Fig. 11, with the increase of heating temperature, the cumulative amplitude of acoustic emission increased or decreased to a small extent, and the cumulative amplitude-heating temperature curve could be approximated as a nearly horizontal trend. By contrast, the amplitude of acoustic emission decreased as a power function with the increase of heating segmented height, indicating that the heating segmented height had a greater impact on the damage characteristics of coal sample.

Figure 12 also showed that with the increase of heating temperature, the increase or decrease of the cumulative ringing count of acoustic emission was also small. It could be approximated that the curve of cumulative ringing count-heating temperature developed in a near horizontal trend, while with the increase of heating segmented height, the cumulative ringing count also decreased in a power function trend. This phenomenon also indicated that the height of heating segmented had a greater influence on the damage characteristics of coal sample.

Further analysis of the relationship between the cumulative amplitude of acoustic emission and the cumulative ringing count and the failure state of coal sample during high-temperature heating could be concluded that the larger the height of the heating segmented was, the more likely coal samples were to be damaged, while the temperature between 100 and 400 °C had little influence on the failure critical state of coal sample. The reason was that the temperature between 100 and 400 °C did not reach the ignition point of the hard coal used in the test, which could not promote the essential change of the coal body. However, the heating process was accompanied by the transfer of heat energy, which would still reduce the heating segmentation's water and gas content. As a result, the internal porosity of the coal sample was increased, which leads to the coal sample being drier and more prone to brittle fracturing. During the application of axial load, the number of acoustic emissions increased correspondingly, and the accompanying energy released by a single acoustic emission also increased correspondingly. As a result, the cumulative amplitude and ringing count of acoustic emission decreased with the increase of heating segmented height.

### Exploration on the inversion of the intensity of coal sample by acoustic emission parameters

Axial compression load was applied after segmented high-temperature treatment of coal sample, and it was found that the height of segmented heating played an important role in the damage and failure characteristics of coal sample. By fitting the laws between the compressive strength, cumulative acoustic emission amplitude, cumulative ringing count and segmented heating height of coal sample, it was found that they all decreased in a power function trend with the increase of segmented heating height. Based on this trend, the relationship between the compressive strength of the coal sample and the cumulative acoustic emission amplitude and ringing count was established. The critical state of coal sample destruction could be inverted by acoustic emission parameters through the proposed relationship.

Based on the phenomenon that the compression strength, cumulative acoustic emission amplitude and ringing count of coal sample all decreased in the form of a power function with the increase of the height of the heating segmented of coal sample, the relationship between each data could be expressed as follows:

1 $$\left\{ \begin{gathered} \sigma_{c} = k_{c} n^{{b_{c} }} \hfill \\ A = k_{1} n^{{b_{1} }} \hfill \\ N = k_{2} n^{{b_{2} }} \hfill \\ \end{gathered} \right.\;\;\;\;\;\left( {{\text{Only}}\;{\text{ relations }}\;{\text{among}}\;{\text{ data}}} \right)$$

where: *σ*_c_ is the compressive strength data of the coal sample; *A* is the acoustic emission amplitude data; *N* is the ringing count data; *k*_c_, *k*_1_, *k*_2_ are the coefficients; *b*_c_, *b*_1_, *b*_2_ are the powers, and all the data in the formula are assumed to be dimensionless.

Further collating formula (1), only considering the relationship among compressive strength data, acoustic emission amplitude data and cumulative ringing count data, the relationship among the three could be expressed as follows:

2 $$\sigma_{c} = aA + bN + C$$

where: *a* and *b* are the coefficients, *C* is the temperature trimming constant, and the data in the formula are assumed to be dimensionless.

The acoustic emission amplitude *A* and cumulative ringing count *N* in formula (2) could be directly measured by the acoustic emission test, while the coefficients *a*, *b* and temperature trimming constant *C* need to be calculated on the trial basis.

**Figure 13:**
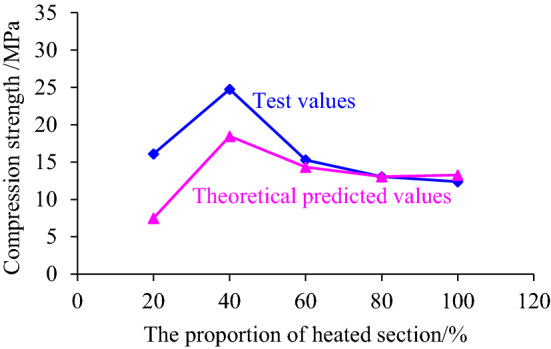
Verification of acoustic emission parameters reflecting coal sample strength (Heating temperature is 200 °C).

Based on the above ideas, the compressive strength and acoustic emission related parameters of coal sample at the heating temperature of 200 °C were selected for verification. The relevant parameters obtained from the test were as follows:

The data in Table 2 were brought into the formula (2) to obtain five equations, and then randomly combined two sets of equations into a system equation groups. To ensure that each equation had solution, first assuming hypothesis temperature constant was zero and obtaining ten solutions of coefficient *a* and *b* by solving equations, the value of *a* and *b* could be obtained by taking the average value − 60.68 and 618.83, respectively. To determine the value of temperature constant *C*, substituting the solved coefficients *a* and *b* and relevant parameters in Table 2 into formula (2), the equation can be formed and the average value of the constant *C* can be calculated. To verify the rationality of the inversion of acoustic emission parameters of coal sample strength, the experimental data, and theoretical prediction data were compared, as shown in Fig. 13.

**Table 2:** Correlative parameters required for acoustic emission parameter inversion of coal sample strength.

Heating section height H_f_/mm	Compression strength *σ*_c_/MPa	Cumulative amplitude *A*/dB	Ringing count/time
20	16.11	1696.04	129.98
40	24.75	1408.97	190.33
60	15.29	460.37	63.99
80	13.04	892.56	96.15
100	12.38	716.06	80.46

Figure 13 showed that the experimental value and the theoretically predicted value changed with the increase of the height of heating segmented in the same trend, and the data were relatively consistent. Although the theoretically predicted value was generally smaller than the experimental value, it still offers reference on predicting the critical failure state of the coal sample subjected to axial load after segmented high-temperature treatment. Therefore, it could be considered that to a certain extent, the cumulative amplitude of acoustic emission and the ringing count could invert the compressive strength data of the coal sample treated by segmented high temperature, which could be used to predict the failure condition of the coal sample under the corresponding conditions.

## Failure mode of coal sample

**Figure 14:**
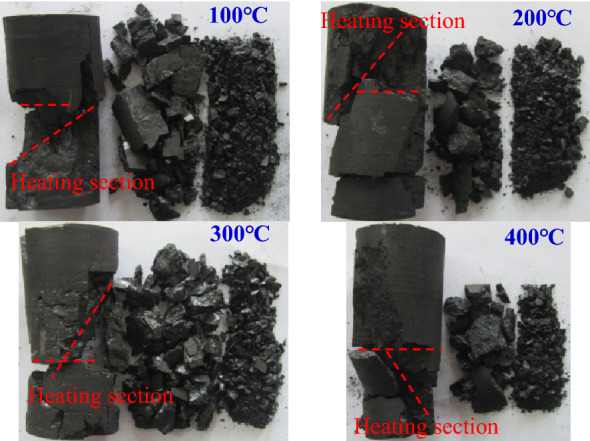
Failure mode when 40% of the height of the coal sample were heated.

**Figure 15:**
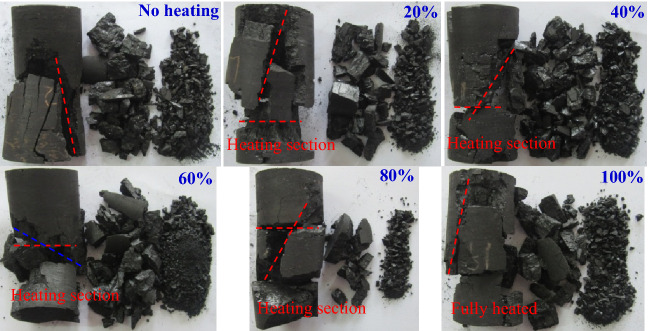
Failure mode of coal sample segmented heating to 300 °C.

The height of heating segmented and heating temperature would change the moisture content and gas content inside the coal sample, thus affecting the final failure mode of the coal sample under axial load. The analysis of the final failure mode of the coal sample could also indirectly reflect the force history of coal sample and the law of acoustic emission generated during the stress. Figures 14 and 15 showed the typical failure modes of coal samples with different heating temperatures and different heating segmented heights, respectively.

Figure 14 showed that when the segmented heating height was constant and the heating temperature increased, the coal samples were mainly damaged by single-incline shear and separation layer failure, and also with a certain degree of friction failure, indicating that the failure modes of coal samples were relatively complex when axial load were applied after heating, but there was no obvious difference in the failure mode of coal samples at different temperatures. The conclusion that heating temperature of 100 °C ~ 400 °C had no dominant effect on the failure mode of coal sample, which was consistent with the previous conclusion that the heating temperature of 100 °C ~ 400 °C had no major effect on the mechanical strength and acoustic emission law of coal sample. By analyzing the location of shear failure and separation layer failure, it was found that the shear surface originated from the heating section, and the separation layer surface was near the interface between the heating and unheated sections. This phenomenon further indicated that the height of heating section changed the form and location of coal sample failure to a certain extent, especially causing the coal sample to produce separation layer failure. Further analysis of the lumpiness of destroyed coal sample could be divided into three levels: large lumpiness, small particles, and crushed particles, of which large lumpiness account for the largest proportion, followed by small particles. The generation of large lumpiness caused shear failure and separation layer failure of coal sample, the existence of small particles was the result of compression-shear fracture when coal strips were formed by separation layer of coal sample, while the crushed blocks were caused by friction between damaged coal blocks.

When the heating temperature was constant and the heating segmented height increased, the locations of shear failure and separation layer failure of coal sample changed, and the degree of failure also increased gradually, as shown in Fig. 15. When the coal sample was not heated, it was mainly two large pieces of failure, and the single-incline shear failure occurred. When the proportion of heating section height to the total height of the coal sample increased from 20 to 100%, the number of large damaged pieces increased from 3 to 5, and all of them produced inclined plane shear failure and separation layer failure. Meanwhile when the height of heating segmented was small, the sheer slope was located in the unheated section. For example, when the height of the heating segmented accounted for 20% of the height of coal sample, and the height of the heating segmented was large, the sheer slope gradually moved to the heating section, indicating that the existence of the heating section changed the trend of micro-cracks derivation inside the coal sample to a certain extent. Further analysis of the location of coal sample separation failure, it was found that it also changed with the increase of the height of heating section, and mostly appeared near the contact surface between the unheated section and the heating section, indicating that the coal body at the contact point of the two was subjected to the thermal effect and produced the tensile expansion effect, which leading to the generation of separation cracks. Analyzing the lumpiness of the damaged coal sample, it could also be divided into three levels: large fragmentation, small single piece, crushing grain of block. Among them, the proportion of large fragmentation was the largest, followed by the small particles, and the crushed particles were the smallest. This phenomenon further indicating that the shear failure and separation layer failure were dominant in the axial loading coal sample after high-temperature treatment, and the mixed friction failure was supplementary.

## Conclusions

Deep hard coal samples were heated by the coal sample segmented heating device prepared by ourselves, and loading mechanical tests were carried out after segmented high-temperature treatment to study the effects of heating temperature and segmented height on the strength, deformation, acoustic emission characteristics and failure mode of deep hard coal. The corresponding conclusions are as follows:

1. After the deep hard coal was subjected to segmented high-temperature treatment when the temperature was less than 400 °C, its compression stress–strain curve went through four stages (i.e., compaction-elasticity-plasticity-failure). The higher the heating section height, the longer the compression stage of the curve.
2. The temperature of 10 °C ~ 400 °C did not change the main component of coal sample, and the temperature below 400 °C had less effect on the elastic modulus, compression strength, and strain corresponding to compression strength of coal. However, the higher the heating section height, the more moisture and gas dissipated inside the coal sample, resulting in lower elastic modulus and compression strength of coal sample. Meanwhile, the increase of the fracture of separated layer in the heating section caused the increasing strain corresponding to compression strength.
3. The acoustic emission characteristics of the segmented heating coal samples were not greatly affected by the temperature of 100–400 °C, but the cumulative acoustic emission amplitude and ringing count decreased as a power function with the increase of heating segmented height. Based on this rule, the inverse formula of the acoustic emission parameters of coal sample strength was established and verified.
4. When subjected to axial load, the segmented heating coal samples, mainly show the inclined plane and separation layer failure, and the friction mixed failure was supplementary. Overall, the failure degree was serious with the increase of heating segmented height.

## Data Availability

The data used to support the findings of this study are included within the article.
